# Serum Amyloid Biomarkers, Tau Protein and YKL-40 Utility in Detection, Differential Diagnosing, and Monitoring of Dementia

**DOI:** 10.3389/fpsyt.2021.725511

**Published:** 2021-09-13

**Authors:** Karolina Wilczyńska, Mateusz Maciejczyk, Anna Zalewska, Napoleon Waszkiewicz

**Affiliations:** ^1^Department of Psychiatry, Medical University of Białystok, Białystok, Poland; ^2^Department of Hygiene, Epidemiology and Ergonomics, Medical University of Białystok, Białystok, Poland; ^3^Experimental Dentistry Laboratory, Medical University of Białystok, Białystok, Poland

**Keywords:** Alzheimer's dementia (AD), vascular dementia (VaD), mixed dementia, amyloid beta, tau protein, YKL-40 (chitinase 3-like 1), serum

## Abstract

**Introduction:** The diagnosis and treatment of dementia is one of the greatest challenges in contemporary health care. The widespread use of dementia biomarkers would improve the quality of life of patients and reduce the economic costs of the disease. The aim of the study was to evaluate the usefulness of proteins related to the Alzheimer's disease pathogenesis—amyloid beta isoform (Aβ) and total tau protein (t-tau), as well as the quite recently discovered marker YKL-40 in the most common types of dementia.

**Methods:** 60 dementia (AD—Alzheimer's disease, VaD—vascular dementia, MxD—mixed dementia) and 20 cognitively normal subjects over 60 years old were examined. Subjects with dementia of etiology different than AD or VaD and with neoplastic or chronic inflammatory diseases were excluded. Concentrations of Aβ40, Aβ42, t-tau, and YKL-40 were measured in serum using ELISA kits on admission and after 4 weeks of inpatient treatment. ANOVA and Tukey's test or Dunn's test were used to perform comparison tests between groups. Correlations were measured using Pearson's coefficient. Biomarker diagnostic utility was assessed with ROC analysis.

**Results:** YKL-40 differentiates between cognitively normal and mild dementia patients with 85% sensitivity and specificity and t-tau with 72% sensitivity and 70% specificity. YKL-40 and t-tau concentrations correlate with each other and with the severity of clinically observed cognitive decline.

**Conclusions:** YKL-40 is a sensitive and specific biomarker of early dementia and, to a lesser extent, of dementia progression, however, many comorbidities may influence its levels. In such conditions, less specific but still reliable t-tau may serve as an alternative marker. Obtained results did not confirm the diagnostic utility of amyloid biomarkers.

## Introduction

Dementia is a group of diseases causing cognitive dysfunction and disturbing functioning in everyday life (1). According to World Health Organization, around 50 million people in the world have dementia and every year there are nearly 10 million new cases (2).

The most common cause of dementia is Alzheimer's disease (AD), found in 60–80% of dementia cases. AD is a primary degenerative disease resulting from the accumulation of amyloid beta (Aβ) plaques in the perineural spaces and fibrillary tangles composed of tau protein inside neurons, leading to nerve cell damage and death. In about half of AD patients, another predominantly vascular cause of dementia coexists, which is classified as mixed dementia (MxD). Vascular dementia (VaD) is the second most common cause of dementia and it accounts for 10% of total dementia cases, while in another 30% it is a component of MxD (1).

The diagnosis of dementia is often made too late due to the insufficient availability of specialistic healthcare and lack of training of personnel (3). To date, the use of AD biomarkers is limited by their high cost, low availability, and invasiveness of the CSF collection procedure (3). A diagnostic test based on a serum biomarker could be widely used, as blood collection is cheaper, faster, less invasive, and more acceptable for the patient (4). An ideal biomarker should give reliable and reproducible results, be inexpensive and easy to use (5), and be related to the neuropathology of the disease, and validated in neuropathologically confirmed cases. It should also be detectable in the early stages of dementia and not be affected by applied treatment. It has been considered that an acceptable level of sensitivity and specificity of the AD biomarker is >85% (4). Biomarkers may also be used to assess the likelihood of preclinical disease occurrence and further prognosis, differential diagnosis, therapeutic response, or disease progression (4). In the process of drug development, biomarkers could help in subject selection and group assignment, as well as in the study drug evaluation (5).

Due to their key role in the pathogenesis of AD, amyloid markers and tau protein are considered as potential biomarkers of dementia in serum. Aβ1-42 is the major component of amyloid plaques, negatively correlating with the burden of amyloid deposits in the brain tissue, while Aβ1-40 is a more soluble, less amyloidogenic form which may even protect against Aβ deposition (5). Amyloidopathy also occurs in VaD as cerebral amyloid angiopathy (6). Some studies have yet confirmed the correlation between blood Aβ1-42 and Aβ1-40 concentrations and the presence of AD (7–9), while the results of other studies contradicted this thesis (10–12). The concentrations of amyloid markers in vascular dementia have been also investigated (13).

The second key element of AD pathogenesis is tauopathy (5). Some studies have confirmed the increase of total tau protein (t-tau) in the serum of AD (14–16) and FTD subjects (17). In a study including a VaD group, the highest t-tau concentrations were obtained in AD, an intermediate in VaD, and the lowest in controls (13).

An inflammatory marker YKL-40 is also considered as a potential biomarker of dementia (18), neoplastic diseases, and chronic inflammation (19). The increase in YKL-40 concentration in AD results from the activation of proinflammatory cells due to cell death caused by the accumulation of beta amyloid (20). Therefore far, the increase in the concentration of this marker in AD has been confirmed in two cross-sectional studies (21, 22).

## Materials and Methods

### Participants

Subjects with dementia were recruited among patients of the psychiatric hospital in Choroszcz, Poland. All subjects gave their informed consent before participation in the study. The study was conducted in accordance with the Declaration of Helsinki, and the protocol was approved by the Ethics Committee of Medical University of Białystok (R-I-002/62/2016).

Examined subjects were hospitalized for psychiatric reasons. Patients with more severe cognitive decline were hospitalized for disorders associated with dementia (e.g., behavioral symptoms), while those with mild dementia—for in-depth neurocognitive diagnosis or for other mental disorders (e.g., anxiety disorders). First, they were prescreened with Mini Mental State Examination (MMSE) according to the PAR MMSE Clinical Guide Reorder #RO-4922. The cut-off MMSE score was set on 23 points. After prescreening, 100 demented patients without inflammatory and neoplastic comorbidities were included.

Subsequently, to exclude secondary dementia, all of them underwent: brain computed tomography and blood tests: morphology, sodium, potassium, chloride, total calcium, urea, creatinine, aspartate aminotransferase (AST), alanine aminotransferase (ALT), C-reactive protein, thyroid stimulating hormone, folic acid, and vitamin B12. In all subjects, laboratory test results were either within the normal range or showed slight, clinically insignificant deviations from the reference values (Supplementary Table 1). Due to the possibility of a decrease in cognitive functioning secondary to depression, the presence of depression was excluded using the Geriatric Depression Scale (GDS).

The diagnosis of dementia subtypes was established clinically by experienced clinical psychologists and psychiatrists based on the case history, observation, computed tomography (presence or absence of vascular lesions in the central nervous system) and a battery of neuropsychological tests selected individually for each patient. The tests were matched to the individual level of cognitive functioning and included tools such as ACE-R, Verbal Fluency Test, Frontal Assessment Battery, Stroop test, Rey Complex Figure Test, Rey Auditory Verbal Learning Test, Trail Marking Test. Patients with clinical features of primary dementia other than AD, such as Lewy body dementia or FTD, were not eligible for the study. The final diagnosis was made based on ICD-10 research criteria [Table 1; (23)].

**Table 1:** ICD-10 dementia research diagnostic criteria used in the study (23).

**DEMENTIA**		
G1. Evidence of each of the following:		
(1) A decline in memory, which is most evident in the learning of new information, although in more severe cases, the recall of previously learned information may be also affected. The impairment applies to both verbal and non-verbal material. The decline should be objectively verified by obtaining a reliable history from an informant, supplemented, if possible, by neuropsychological tests or quantified cognitive assessments. For example, the individual has difficulty in registering, storing and recalling elements in daily living, such as where belongings have been put, social arrangements, or information recently imparted by family members.		
(2) A decline in other cognitive abilities characterized by deterioration in judgement and thinking, such as planning and organizing, and in the general processing of information. Evidence for this should be obtained when possible from interviewing an informant, supplemented, if possible, by neuropsychological tests or quantified objective assessments. Deterioration from a previously higher level of performance should be established. Activities are increasingly restricted and poorly sustained.		
G2. Preserved awareness of the environment [i.e., absence of clouding of consciousness (as defined in F05, criterion A)] during a period of time long enough to enable the unequivocal demonstration of G1.		
G3 A decline in emotional control or motivation, or a change in social behavior, manifest as at least one of the following: (1) emotional lability; (2) irritability; (3) apathy; (4) coarsening of social behavior.		
G4. For a confident clinical diagnosis, G1 should have been present for at least 6 months; if the period since the manifest onset is shorter, the diagnosis can only be tentative.		
**AD (F00)**	**VaD (F01)**	**MxD (F00.2)**
A. The general criteria for dementia (G1 to G4) must be met. B. There is no evidence from the history, physical examination, or special investigations for any other possible cause of dementia (e.g., cerebrovascular disease, Parkinson's disease, Huntington's disease, normal pressure hydrocephalus), a systemic disorder (e.g., hypothyroidism, vit. B12 or folic acid deficiency, hypercalcaemia), or alcohol- or drug-abuse.	G1. The general criteria for dementia (G1 to G4) must be met. G2. Unequal distribution of deficits in higher cognitive functions, with some affected and others relatively spared. Thus memory may be quite markedly affected while thinking, reasoning and information processing may show only mild decline. G3. There is clinical evidence of focal brain damage, manifest as at least one of the following: (1) unilateral spastic weakness of the limbs; (2) unilaterally increased tendon reflexes; (3) an extensor plantar response; (4) pseudobulbar palsy. G4. There is evidence from the history, examination, or tests, of a significant cerebrovascular disease, which may reasonably be judged to be etiologically related to the dementia (e.g., a history of stroke; evidence of cerebral infarction).	A. All of the AD criteria, except from the absence of cerebrovascular disease met.B. VaD criteria met.

Of the 100 prescreened subjects, 60 patients with dementia were finally qualified for biomarker determinations, including 20 with AD, 20 with VaD, and 20 with MxD with Alzheimer's and vascular features. The subjects chosen from prescreened group had the lowest burden of comorbidities in order to minimize the possible impact of these diseases and their treatment on the study results. Assessment of the MMSE scale was performed by the same investigator twice (at the beginning and after 4 weeks of treatment) in the study group. Raw MMSE results were adjusted for age and education level of examined subjects using the formula: adjusted MMSE = raw MMSE – (0.471 × [Education-12]) + (0.131 × [Age-70]) (24). The blood for biomarker determination was collected twice—on admission and after 4 weeks of inpatient treatment.

To assess the impact of dementia severity on biomarker concentrations, we also divided the subjects with dementia into two groups. Patients with an adjusted MMSE score ≥20 points were qualified for the mild dementia group (MD) and those with lower MMSE scores for the moderate to severe dementia group (MSD). The MD group contained 17 subjects (4 AD, 7 VaD, and 6 MxD) and the MSD group-−43 subjects (16 AD, 13 VaD, 14 MxD). The demographic characteristics of the groups are shown in Supplementary Table 2.

The control group was consisted of 20 cognitively normal attenders of the Healthy Senior University at the Faculty of Health Sciences of the Medical University of Bialystok. The healthy volunteers had adjusted Mini Mental State Examination (MMSE) test results within the normal range (27 points or more). In the control group, the occurrence of chronic inflammatory and neoplastic diseases was excluded using the medical history and a panel of laboratory tests. As in the study groups, depression was excluded using the GDS scale.

All participants were over 60 years of age.

### Biomarker Determination

Blood was collected from the ulnar vein, centrifuged, and then frozen at −80°C until the biomarker determination was performed. In the study groups, blood was taken twice—on admission and after 4 weeks of hospitalization, to assess the possible impact of the treatment on the concentration of biomarkers. The concentration of YKL-40, t-tau, Aβ1-40, and Aβ1-42 in serum was determined by enzyme immunoassay ELISA using ready-made diagnostic kits from USCN Life Science, Wuhan, China. The manufacturer's instructions were followed. The absorbance of the samples was measured using an Infinite M200 PRO Multimode Microplate Reader (Tecan). All determinations were made in duplicate tests.

### Statistical Analysis

Statistical analysis of the results was performed with GraphPad Prism 7.0 for MacOS (GraphPad Software, La Jolla, USA). The D'Agostino-Pearson test and the Shapiro-Wilk test were used to assess the normality of the distribution, and the Leven test to assess the homogeneity of variance. All data are presented in graphs or tables as mean and standard deviation (SD). For comparisons between groups, ANOVA and Tukey's test were used, and in the absence of normal distribution—the Kruskal-Wallis and Dunn's test ANOVA. Multiplicity adjusted *p*-value was also calculated. Correlations between biomarkers were assessed using the Pearson correlation coefficient. Multivariate analysis of the simultaneous impacts of many independent variables on one quantitative dependent variable was made by means of linear regression. Gender, age, and MMSE were included as independent variables; 95% confidence intervals (CI) were reported along with regression parameters. The diagnostic usefulness analysis of biomarkers was assessed using Receiver Operating Curve (ROC) analysis. The confidence intervals of sensitivity and specificity were calculated using Wilson/Brown method. The level of statistical significance was set at *p* ≤ 0.05.

## Results

### Demographics and Diagnostic Tests

The analysis of variance showed no statistically significant differences in the age and education of the respondents between the groups (Supplementary Table 1). The groups were consisted of 60 women and 20 men (AD - 6; VaD - 4; MxD - 5; C - 5 men).

**Figure 1:**
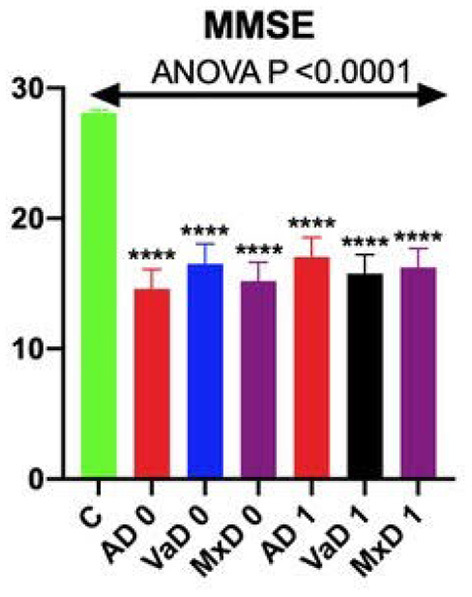
MMSE scores on admission and after 4 weeks of hospital treatment. ****p vs. C <0.0001.

The results of basic examinations and studies aimed at excluding secondary causes of dementia were within the normal range. The only statistically significant differences between the groups were observed in the concentrations of sodium, chloride, MCV, platelecrit, aspartate aminotransferase, and blood glucose (Supplementary Table 1). All subjects of the study group had brain CT scan. Only those without CNS vascular changes were qualified to the AD group. None of the subjects had acute brain hemorrhage or ischemia, tumors, or other lesions that might indicate a cause of dementia other than AD or VaD. The GDS test ruled out depression in all participants. The obtained GDS results ranged 0–6 points. Assessment of the MMSE scale was performed once in the control group and twice (at the beginning and after 4 weeks of treatment) in the study group. The obtained results were comparable in all groups with dementia and statistically significantly lower than in the control group. There were no significant differences in the adjusted MMSE score before and after 4 weeks of inpatient treatment (Figure 1).

### Correlation of Biomarker Concentrations With the Assessed Parameters

YKL-40 correlated with the concentrations of other markers (t-tau and Aβ1-42/Aβ1-40), the severity of dementia as reflected by the MMSE score, and the parameters of inflammation (C-reactive protein and percentage of neutrophils). A negative correlation with ALT activity was also observed.

T-tau correlated positively with YKL-40 and dementia severity (negative correlation with the MMSE score). Among the laboratory parameters, the correlation with the percentage of neutrophils and the concentrations of sodium, calcium, and creatinine achieved the level of statistical significance.

Correlations between the values of the Aβ1-42/Aβ1-40 index and the concentrations of YKL-40 and Aβ1-40 were also observed. There was no relationship between amyloid markers and t-tau concentrations. The concentrations of Aβ1-40 and Aβ1-42 also correlated with the concentration of chloride and the values of some blood count parameters. Statistically significant correlations between biomarkers, MMSE, inflammatory parameters and obtained in statistical analysis are summarized in Table 2. All other statistically significant correlations were put in Supplementary Table 3.

**Table 2:** Statistically significant correlations found between MMSE, inflammatory parameters and analyzed biomarkers.

**Parameters**	***R***	**95% CI**	***p***
YKL-40 0 and YKL-40 1	0.461	0.2342 to 0.6398	< 0.0001
YKL-40 0 and Aβ1-42/Aβ1-40 0	0.288	0.03707 to 0.5052	0.026
YKL-40 0 and MMSE 0	−0.614	−0.7507 to −0.4263	< 0.0001
YKL-40 1 and t-tau 1	0.36	0.1163 to 0.5622	0.005
YKL-40 1 and MMSE 1	−0.563	−0.7145 to −0.3601	< 0.0001
YKL-40 1 and CRP	0.29	−0.2512 to 0.2566	0.025
YKL-40 1 and Neu	0.507	0.2861 to 0.6767	< 0.0001
t-tau 0 and MMSE 0	−0.287	−0.5041 to −0.03550	0.026
t-tau 1 and MMSE 1	−0.287	−0.5041 to −0.03551	0.026
t-tau 1 and Neu	0.269	0.01127 to 0.4929	0.041
Aβ1-40 0 and Aβ1-40 1	0.323	0.07473 to 0.5328	0.012
Aβ1-40 0 and Aβ1-40/Aβ1-42 0	−0.819	−0.8881 to −0.7131	< 0.0001
Aβ1-40/AB1-42 1 and Aβ1-40 1	−0.778	−0.2503 to 0.2575	< 0.0001
MMSE 0 and CRP	−0.407	−0.5992 to −0.1710	0.001
MMSE 0 and Neu	−0.674	−0.7937 to −0.5027	< 0.0001

*MMSE, Mini Mental State Examination; CRP, C-reactive protein; Neu, neutrophils; Leu, leucocytes; YKL-40 0, YKL-40 concentration on admission; YKL-40 1, YKL-40 concentration after 4 weeks of treatment; MMSE 0, MMSE on admission etc*.

### Differences in Biomarker Concentrations in Various Types of Dementia

**Figure 2:**
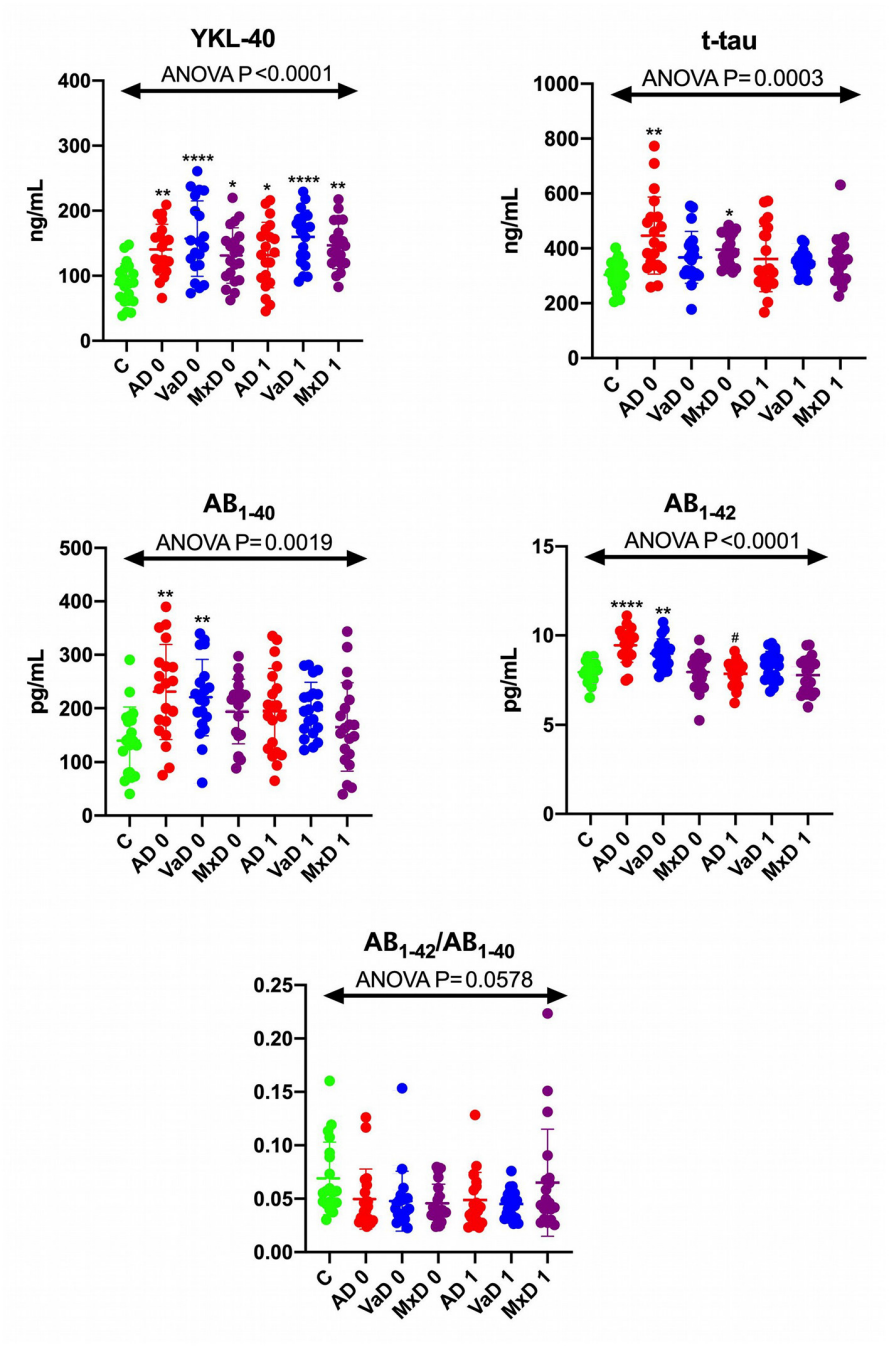
Biomarker concentrations in control and dementia patients on admission and after 4 weeks of treatment. Results are presented as mean, standard deviation (SD) and individual values. C, control group (*n* = 20); AD 0, patients with Alzheimer's dementia on admission (*n* = 20); VaD 0, patients with vascular dementia on admission (*n* = 20); MxD, patients with mixed dementia on admission (*n* = 20); AD 1, patients with Alzheimer's dementia after 4 weeks of treatment (*n* = 20); VaD 0, patients with vascular dementia after 4 weeks of treatment (*n* = 20); MxD, patients with mixed dementia after 4 weeks of treatment (*n* = 20). *p < 0.05 vs. C, **p < 0.005 vs. C, ****p < 0.0001 vs. C, ^#^*p* < 0.05 vs. AD 0.

Figure 2 shows the biomarker concentrations in controls and in demented subjects on admission and after 4 weeks of treatment. YKL-40 concentrations in all dementia types, both on admission and after 4 weeks, were statistically significantly higher than in the control group. The concentrations of other biomarkers were higher only on admission. Significantly higher concentrations of t-tau were observed in AD and MxD.

Aβ1-40 and Aβ1-42 were increased in AD and VaD, which is not surprising considering that amyloidopathy occurs in both dementia types. The Aβ1-42/Aβ1-40 ratio did not differ significantly between the groups.

The ROC analysis (Table 3) indicates a high sensitivity and specificity (70–85%) of YKL-40 in the differentiation between dementia and the control group, however, it does not confirm the utility of YKL-40 in differentiating dementias of various etiologies. YKL-40 is the most diagnostic for AD (sensitivity 85%, specificity 75%, AUC 0.8625) and VaD (sensitivity and specificity more than 80%, AUC 0.8525), for MxD (sensitivity and specificity ~70% each, AUC 0.7975).

**Table 3:** ROC analysis of serum YKL-40 in different types of dementia.

	**AUC**	**95% CI**	***P***	**Cut-off**	**Sensitivity%**	**95% CI**	**Specificity%**	**95% CI**
**t-tau**								
C vs. AD 0	0.845	0.7188–0.9712	0.0002	>338.4	75	53.13–88.81%	70	48.10–85.45%
C vs. VaD 0	0.7175	0.5561–0.8789	0.0186	>318.3	60	38.66–78.12%	55	34.21–74.18%
C vs. MxD 0	0.8825	0.7825–0.9825	<0.0001	>341.4	75	53.13–88.81%	70	48.10–85.45%
AD 0 vs. VaD 0	0.675	0.5060–0.8440	0.0583	<380.4	65	43.29–81.88%	65	43.29–81.88%
AD 0 vs. MxD 0	0.5975	0.4131–0.7819	0.2914	<394.4	55	34.21–74.18%	55	34.21–74.18%
VaD 0 vs. MxD 0	0.645	0.4675–0.8225	0.1167	>375.1	60	38.66–78.12%	60	38.66–78.12%
**YKL-40**								
C vs. AD 0	0.8625	0.7510–0.9740	<0.0001	>106.3	85	63.96–94.76%	75	53.13–88.81%
C vs. VaD 0	0.8525	0.7352–0.9698	0.0001	>113.0	80	58.40–91.93%	80	58.40–91.93%
C vs. MxD 0	0.7975	0.6621–0.9329	0.0013	>103.9	70	48.10–85.45%	70	48.10–85.45%
AD 0 vs. VaD 0	0.5750	0.3926–0.7574	0.4171	>139.8	55	34.21–74.18%	50	29.93–70.07%
AD 0 vs. MxD 0	0.5775	0.3984–0.7566	0.4017	<130.7	50	29.93–70.07%	50	29.93–70.07%
VaD 0 vs. MxD 0	0.6200	0.4436–0.7964	0.1941	<137.9	55	34.21–74.18%	55	34.21–74.18%

T-tau was diagnostic for AD and MxD with 75% sensitivity and 70% specificity (AUC 0.845 and 0.8825, respectively), while its diagnostic value in VaD turned out to be poor.

### Changes in Biomarker Concentration Over Time

The distribution of concentrations of individual biomarkers determined at the beginning of hospitalization and after 4 weeks of its duration is presented in Figure 2. Aβ1-42 concentrations in patients with AD declined after 4 weeks of treatment. In the remaining cases, the concentrations of biomarkers did not change significantly over time.

### Biomarkers in the Assessment of Dementia Progression

All subjects, regardless of the etiology of dementia, were divided into mild dementia (MD) and moderate to severe dementia (MSD) groups, taking the MMSE score of 20 as the cut-off point. The results of ROC analysis for individual markers are summarized in Table 4 (t-tau and YKL-40) and Supplementary Table 4 (amyloid markers). The highest sensitivity and specificity in differentiating between healthy subjects and patients with mild dementia was obtained for YKL-40 (sensitivity 86.05%, specificity 85%, AUC 0.9128) and slightly lower for t-tau (72.09%, 70% and 0.8488, respectively). The differences between the concentrations of amyloid markers were also statistically significant, while their sensitivity and specificity were lower than for YKL-40 and t-tau, and the AUC values were below 0.8.

**Table 4:** ROC analysis of serum t-tau and YKL-40 compared between mild dementia (MD), moderate to severe dementia (MSD), and control group.

**Comparison**	**AUC**	**95% CI**	***P***	**Cut-off**	**Sensitivity%**	**95% CI**	**Specificity%**	**95% CI**
**t-tau**								
C vs. MD	0.8488	0.7553–0.9423	<0.0001	>341.4	72.09	57.31–83.25%	70	48.10–85.45%
C vs. MSD	0.7294	0.5613–0.8975	0.0174	>324.8	64.71	41.30–82.69%	65	43.29–81.88%
MD vs. MSD	0.6607	0.5114–0.8101	0.0539	<377.9	58.82	36.01–78.39%	58.14	43.33–71.62%
**YKL-40**								
C vs. MD	0.9128	0.8412–0.9843	<0.0001	>115.8	86.05	72.74–93.44%	85	63.96–94.76%
C vs. MSD	0.6471	0.4692–0.8249	0.1276	>90.60	52.94	30.96–73.83%	55	34.21–74.18%
MD vs. MSD	0.8263	0.7067–0.9458	<0.0001	<123.3	76.47	52.74–90.44%	76.74	62.26–86.85%

In the comparison between MD and MSD, only YKL-40 proved to be diagnostic for dementia progression. At the cut-off point of 123.3 ng/ml, the sensitivity was 76.47%, specificity-−76.74%, and the AUC was 0.8263. The sensitivity and specificity in differentiating the stages of dementia for the remaining biomarkers ranged from 50 to 60%, and the areas under the curve were below 0.7.

Aβ1-40 has shown the ability to differentiate between controls and subjects with more severe dementia, however, its ROC parameters do not show its utility in discrimination between cognitively normal and subjects with mild dementia, as well as between dementia stages (Table 4).

### Multifactorial Regression on the Assessed Biomarkers

The results of regression analysis are presented in Table 5 for t-tau and YKL-40 and in Supplementary Table 5 for amyloid markers. We did not show the influence of age and gender on the evaluated biomarkers.

**Table 5:** Multifactorial regression of YKL-40 and t-tau.

**Dependent variable**	**Independent variable**			
		**Age**	**Sex**	**MMSE**
YKL-40	EE	0.2469	9.532	−4.376
	95%CI	−1.645 to 2.139	−12.19 to 31.25	−5.895 to −2.857
	*P*-value	0.7948	0.3831	<0.0001
t-tau	EE	1.229	−48.13	−4.631
	95%CI	−3.797 to 6.256	−105.8 to 9.569	−8.666 to −0.5963
	*P*-value	0.6261	0.1003	0.0252

Ykl-40 and t-tau, but not amyloid levels, were dependent on dementia severity assessed with MMSE score.

## Discussion

### Clinicians' Expectations Regarding Dementia Biomarkers

Undoubtedly, the significant value of our study is the fact that the qualification of patients and diagnostic procedures were conducted by a clinician treating patients during hospitalization for several weeks, having extensive knowledge about the patient's medical history and staying in touch with their caregivers. In everyday medical practice, the benefits of developing and disseminating easy-to-use diagnostic tests have been recognized many times.

Fifty two out of the 60 patients we studied were diagnosed with dementia for the first time in their lives. Patients with mild dementia were often hospitalized because of other psychopathological symptoms (e.g., anxiety) and subjectively regarded their cognitive functioning as normal. Memory disorders were also sometimes unnoticed by caregivers or considered as a physiological element of the aging process. Dementia was also often not diagnosed and not treated by other specialists taking care of the patient, for example, by primary care doctors. As previously mentioned, this may result from both limited access to health services (count and duration of visits) and insufficient training in diagnosing dementia of the medical staff (3). It also seems that, in the opinion of many physicians, the diagnosis and treatment of dementia makes little sense due to the low effectiveness of the applied therapies and the predicted short survival time. A cheap and readily available screening test would allow the early identification of patients with a high probability of dementia requiring further specialistic diagnosis (3).

In addition to the direct benefits for patients of early detection of dementia and accurate diagnosis of dementia, new biomarkers could also facilitate clinical trials of novel medications. The diagnostic difficulties described above may contribute to the incorrect qualification of some patients for examination and consequently obtaining unreliable results. It is also difficult to properly quantify the severity of dementia in the investigated subjects (5).

The biomarkers currently used in the diagnosis of dementia are amyloid peptides and tau protein in the cerebrospinal fluid or amyloid beta identified in the brain *in vivo* by PET. These tests are expensive, available only in highly specialized centers. Performing a lumbar puncture to collect CSF is associated with the risk of complications (4). The development of diagnostic tests with the use of biomarkers measurable in biological material that can be collected in a simple and minimally invasive way (such as blood) would significantly improve the effectiveness of diagnosis and treatment of dementia.

### YKL-40 as a Marker Identificating Dementia and Determining Its Severity

Thus far, only a few studies of YKL-40 as a dementia biomarker have been conducted (21, 22). The obtained results indicate its potential diagnostic usefulness and encourage further research. The activation of the inflammatory process due to damage to nerve cells by neurodegeneration (AD) or ischemia (VaD) seems to be responsible for the increase in YKL-40 concentrations in dementia (20).

The obtained YKL-40 concentrations were statistically significantly higher in all study groups compared to the control group, and the high diagnostic value was confirmed by the ROC analysis (AUC from 0.8 for MxD to 0.86 for AD). This indicates the potential usefulness of this marker in screening for dementia. We also observed a correlation of YKL-40 concentrations with the level of cognitive functioning measured with the MMSE scale and with t-tau concentrations, which confirms the cohesion of the obtained results.

Sensitivity and specificity at the level of at least 85% suggests that the diagnostic test can be used as a dementia biomarker (4). These criteria were met for the differentiation of the control group from mild dementia. Slightly lower (>76%), but still satisfactory values of those parameters were also obtained when trying to differentiate between mild and moderate to severe dementia with the use of YKL-40 protein. Those results indicate its higher sensitivity and specificity both in the early detection and in monitoring the progression of dementia than amyloid and t-tau.

The most serious limitation of the use of YKL-40 as a biomarker seems to be its non-specificity. Its concentration increases during many diseases, especially inflammatory diseases (including neuroinflammatory processes) and neoplastic diseases, which are quite common comorbidities in the elderly population (19, 20). Therefore, when using YKL-40 as a diagnostic test, it would be necessary to obtain at least an in-depth medical history regarding comorbidities to avoid misinterpretation of the biomarker levels. It might also be useful to determine the concentration of another non-specific marker of the inflammatory response simultaneously. Such a marker could be, for example, the C-reactive protein or the percentage of neutrophils, which showed a positive correlation with the concentration of YKL-40 in our study.

Due to the low serum concentrations (nanograms/milliliter), the accuracy of the quantification of YKL-40 protein may be highly dependent on the laboratory methods used. We decided to determine the concentrations of YKL-40 using the ELISA method due to its relatively low cost and the large number of experienced laboratory diagnosticians.

### T-Tau as an Alternative Dementia Diagnostic Marker

The tau protein, despite its lower sensitivity and specificity, may be an alternative marker to YKL-40 in the diagnosis of early stages of dementia, especially for patients in whom the concentration of YKL-40 is elevated for other reasons, such as chronic inflammatory diseases. The tau protein is a marker that is a characteristic of a group of diseases called tauopathies, most of which are rare diseases or may be easily recognized by their specific symptoms [e.g., Down syndrome; (25)]. It follows that the risk of a falsely positive result due to a coexisting disease is low for t-tau.

The t-tau protein concentrations were the highest in the AD group, a slightly smaller increase was observed in MxD, and the marker concentration in the VaD group did not differ from that observed in the control group, which reflects the role of t-tau in the etiology of individual types of dementia. The sensitivity of 72.09% and the specificity of 70% in the diagnosis of early dementia is lower than YKL-40, but still higher than that of amyloid markers, and may be sufficient for t-tau use in brief screening. The reliability of t-tau as a marker of dementia is also supported by the correlation of its concentrations with the results of the MMSE test and YKL-40. It might be surprising that the diagnostic accuracy of t-tau, as well as YKL-40 was better in differentiating between control and mild dementia than between controls and MSD. This may be explained by the results of a study by Llibre-Guerra et al. analyzing the longitudinal changes in AD CSF biomarkers. In this study, the t-tau CSF levels in AD subjects did not increase and p-tau-181 even decreased after the disease onset. The observed biomarker trajectories were consistent with the degree of brain atrophy observed in MRI. No further increase of biomarkers in the later disease stages may be explained by a lesser extent of cellular stress and inflammation and the less number of neuronal tissue and neuronal substrates to produce tau (26).

Due to the common morbidity in geriatric patients, it is important to have a diagnostic test not significantly affected by concomitant diseases. This suggests a possible diagnostic usefulness of t-tau in patients with diagnosed or suspected conditions that may cause an increase of YKL-40 concentration in serum. However, the obtained results indicate the risk of a fairly high percentage of false-positive and false-negative results when using this biomarker. A solution might be to use a combination of two or more biomarkers playing different roles in the pathophysiology of dementia. The results of our study show that t-tau may be a quite reliable marker of AD and MxD with Alzheimer's features, but not necessarily for VaD.

### Serum Amyloid Markers Cannot Be Used as Dementia Markers

Serum amyloid biomarkers, especially Aβ1-42 and Aβ1-40 in serum, were initially considered as potential markers of dementia at the end of the last century (20). This resulted mostly from a previously confirmed increase in the “amyloidogenic” Aβ1-42 isoform at the expense of a decrease in the amount of more soluble Aβ1-40 in the CSF (5). It was expected that similar amyloid isoform concentration changes might be observed in peripheral blood.

In serum, amyloid markers do not correlate with the presence or severity of dementia as measured by the MMSE scale. It may result from the limited and individually variable permeability of amyloid peptides across the blood-brain barrier, as well as from originating some portion of serum Aβ from tissues other than the brain.

We also found no significant differences between the levels of amyloid markers in different types of dementia. This seems understandable due to the presence of amyloidopathy not only in AD, but also in VaD (amyloid angiopathy) (6).

### Biomarkers in the Differential Diagnosis of Dementia

Dementia is a group of diseases of various etiologies, pathophysiology, and prognosis. The effectiveness of the used pharmacological treatment methods is also different depending on the dementia subtype. On the other hand, the pivotal clinical signs are largely common to all types of dementia. Some additional symptoms are specific to particular types of dementia, for example, impaired motor function may indicate vascular dementia, hallucinations, and parkinsonism speak for dementia with Lewy bodies, and aphasia as well as personality changes make frontotemporal dementia plausible (1). However, it is not possible to reliably establish the etiology of dementia on the basis of clinical symptomatology alone (3, 4). The changes observed in brain neuroimaging also do not allow for a clear determination of the type of dementia. For example, in a patient with numerous cerebral vascular lesions, Alzheimer's etiology of dementia cannot be ruled out, firstly because of the possibility of coexistence of both pathophysiological processes, and secondly because of the possibility of vascular pathology secondary to amyloid deposition in the blood vessels. Such a patient could be unjustly disqualified from treatment with a preparation used exclusively in Alzheimer's dementia, losing the potential benefits of pharmacotherapy.

Our results do not indicate the usefulness of any of the studied biomarkers in the differential diagnosis between different types of dementia. The greatest differences were observed in the case of t-tau, the concentration was statistically significantly higher in the AD group, a slightly smaller increase was observed in MxD, and the marker concentration in the VD group did not differ from that observed in the control group, which in a way reflects the role of t- tau in the etiology of those types of dementia. Amyloid beta and YKL-40 are not dementia specific markers, and the increase in their concentrations occurs in many neurinflammatory processes of various etiologies. These observations are consistent with the results of previous studies, which found a quantitatively non-specific increase in the concentration of amyloid and t-tau markers for the type of dementia (18).

### Biomarkers in Monitoring of Treatment Response

The possibility of quantifying the severity of the disease using a biomarker could facilitate the effectiveness assessment of the applied pharmacotherapy, which is particularly important in clinical trials (4). The clinical scales currently used for this purpose may give unreliable results for reasons such as learning questions and tasks repeated at each visit or mental and somatic complaints (e.g., anxiety or concentration on pain). The results may also be inconclusive due to sensory dysfunctions common in old age, such as uncorrected or uncorrectable seeing and hearing defects. The possibility of repeatable, minimally invasive determination of the disease biomarker would also allow for a more precise assessment of the effects of the therapy.

We found a statistically significant decline in the concentration of Aβ1-42 in AD after 4 weeks of treatment. These results are understandable considering the role of amyloid a in dementia/aging/inflammation (5), but were difficult to interpret in the context of the lack of changes in other marker's concentrations. We suspect that Aβ1-42 decline might have occurred due to a general improvement of mental and/or general health after treatment. The lack of improvement in cognitive functioning as measured by the MMSE scale after 4 weeks seems to support this thesis.

### Study Limitations

Clinical diagnoses of dementia subtypes established on the basis of an interview, observation, neuropsychological diagnosis, and exclusion of the most common secondary causes of dementia are uncertain to at some extent. Diagnostics based on the examination of amyloid markers and/or tau protein in the cerebrospinal fluid or the use of PET examination would be much more reliable, but at the same time a costly method of qualifying patients with AD. Moreover, for economic reasons, the subjects underwent brain imaging using computed tomography, instead of the more accurate and more recommended magnetic resonance imaging.

The method of assessing the severity of dementia that we have adopted may also have some limitations. We based it on the MMSE test results adjusted for the age and education level of the respondents. MMSE is a quick and easy test, and the availability of its validated language version makes it possible to compare the results of studies conducted on different populations in various languages. On the other hand, its serious disadvantage is the fact that it primarily assesses the efficiency of memory, and to a lesser extent other cognitive functions, but does not take into account other symptoms accompanying cognitive deficits. In practice, it is often observed that patients with similar results of cognitive tests may present diametrically different levels of functioning, which makes it difficult to quantify the severity of dementia. The refinement of methods to quantify dementia progression could improve the quality of future research on this clinical syndrome.

As mentioned above, in our study there is a lack of validated biomarkers (e.g., amyloid PET, CSF, and/or FDG-PET) to support the diagnosis of different types of dementia and the lack of a comparable neuropsychological evaluation to assess cognitive impairment. However, the selection of neuropsychological tests and the diagnosis of individual patients were based on the profound clinical experience of a neuropsychologist who evaluated all patients.

Another important limitation of our study is the sample size. Due to the promising results we obtained, we believe that the study is worth to be repeated on a larger group of respondents.

### Future Directions

More research is needed to enable long-term follow-up in dementia patients. We are convinced that long-term prospective study on serum dementia biomarkers would bring valuable data that might contribute to the development of dementia screening and follow-up markers.

Finally, there is also a need to look for new biomarkers, especially those that would contribute to a non-invasive diagnosis of dementia. Especially promising are salivary redox parameters, which with high sensitivity and specificity differentiate patients with mild dementia from severe dementia as well as AD from VaD and MxD (27, 28). Saliva can be a promising, easily accessible, and non-expensive diagnostic material used in patients with neuropsychiatric disorders (29, 30). There are also some promising blood biomarkers some tau isoforms, such as p-tau-217 (18). Nevertheless, further studies are required on a large population of patients.

## Conclusions

YKL-40 is a highly sensitive and specific marker that differentiates healthy individuals from patients with Alzheimer's, vascular or mixed dementia. It is particularly sensitive and specific in the diagnosis of dementia onset, and slightly less in the assessment of progression to more advanced stages of the disease. The possibilities of using YKL-40 as a biomarker are limited by its non-specificity. T-tau shows a slightly lower but still satisfactory sensitivity and specificity in differentiating between mild AD, MxD, and the control group. T-tau may be a marker particularly useful for the diagnosis of patients with coexisting diseases associated with an increase of YKL-40 concentrations in serum.

Our study results do not indicate the diagnostic usefulness of amyloid markers (Aβ1-40, Aβ1-42, and Aβ1-42/Aβ1-40). The results do not indicate the usefulness of any of the examined biomarkers in the differential diagnosis between dementias of different etiologies. Decreased concentrations of Aβ1-42 in AD after 4 weeks of inpatient treatment might be due to the improved general and/or mental health while treatment/hospitalization.

## Data Availability Statement

The raw data supporting the conclusions of this article will be made available by the authors, without undue reservation.

## Ethics Statement

The studies involving human participants were reviewed and approved by Ethics Committee of Medical University of Białystok. The patients/participants provided their written informed consent to participate in this study.

## Author Contributions

NW and KW: conceptualization, resources, writing—review and editing, project administration, and funding acquisition. NW, AZ, MM, and KW: methodology. MM: software. MM and AZ: validation. KW, MM, and NW: formal Analysis. KW and MM: investigation, data curation, and visualization. KW: writing—original draft preparation. NW and AZ: supervision. All authors contributed to the article and approved the submitted version.

## Funding

MM was supported by the Foundation for Polish Science (FNP).

## Conflict of Interest

The authors declare that the research was conducted in the absence of any commercial or financial relationships that could be construed as a potential conflict of interest.

## Publisher's Note

All claims expressed in this article are solely those of the authors and do not necessarily represent those of their affiliated organizations, or those of the publisher, the editors and the reviewers. Any product that may be evaluated in this article, or claim that may be made by its manufacturer, is not guaranteed or endorsed by the publisher.
