# Supplementary material for: Serum Amyloid Biomarkers, Tau Protein and YKL-40 Utility in Detection, Differential Diagnosing, and Monitoring of Dementia
Source: Front Psychiatry. 2021 Sep 13;12:725511. doi: 10.3389/fpsyt.2021.725511 (PMC8473887; doi:10.3389/fpsyt.2021.725511)
Supplement: Supplementary file 1 [file Data_Sheet_1.docx]

Supplementary Material

# Supplementary table 1. Participant characteristics (p<0.005: a – vs C; b – vs MxD).

| Parameter | C (N=20) | AD 0 (N=20) | VaD 0 (N=20) | MxD 0 (N=20) | ANOVA p-value |
| --- | --- | --- | --- | --- | --- |
| Age [years] | 74.15±5.706 | 78.55±6.549 | 77.95±5.125 | 78.1±5.004 | 0.0562 |
| Education [years] | 6.894±1.094 | 6.35±3.249 | 7.4±2.624 | 5.7±2.716 | 0.1876 |
| Leukocytes [10^3^/μl] | 7.513±1.114 | 8.09±2.272 | 7.6±2.473 | 6.61±1.258 | 0.0953 |
| Lymphocytes [%] | 25.15±4.899 | 22.21±7.794 | 26.82±8.167 | 27.08±11.37 | 0.2481 |
| Monocytes [%] | 6.947±1.47 | 7.165±1.977 | 7.967±1.383 | 8.27±4.072 | 0.2937 |
| Neutrophiles [%] | 64.18±5.606 | 68.96±7.437 | 63.02±8.72 | 63.06±12.44 | 0.1252 |
| Eosynophiles [%] | 1.983±0.08617 | 1.305±1.285 | 1.8±1.262 | 1.27±0.6284 | 0.0447 |
| Basophiles [%] | 0.4389±0.1111 | 0.365±0.1872 | 0.3667±0.191 | 0.33±0.2677 | 0.3659 |
| Erythrocytes [10^6^/μl] | 4.576±0.5064 | 4.553±0.5315 | 4.676±0.4325 | 4.458±0.508 | 0.6062 |
| Hemoglobin [g/dl] | 13.45±0.6836 | 13.67±1.671 | 14.04±1.239 | 13.86±1.021 | 0.4706 |
| Hematocrit [%] | 41.95±3.108 | 41.43±5.673 | 41.81±3.535 | 41.82±3.825 | 0.9818 |
| Mean corpuscular volume [fl] | 91.82±3.225 | 90.65±4.427 | 89.49±3.437 b | 94.22±6.26 | 0.0129 |
| Mean cell hemoglobin concentration [g/dl] | 32.06±0.7749 | 31.98±2.219 | 32.19±1.806 | 32.72±1.763 | 0.527 |
| Red cell distibution width [%] | 14.79±0.9156 | 14.75±1.234 | 14.55±0.8155 | 15.24±1.955 | 0.43 |
| Platalets [10^9^/μl] | 248.1±55.48 | 314.2±130.9 | 263.4±83.88 | 271.1±81.76 | 0.1422 |
| Mean platelet volume [fl] | 8.089±1.18 | 8.175±0.921 | 8.072±0.9028 | 8.12±0.9059 | 0.9889 |
| Platelecrit [%] | 0.2625±0.04611 | 0.2533±0.09755 | 0.2087±0.05702 | 0.2158±0.05155 | 0.0303 |
| Platelet distibution width [%] | 13.66±1.099 | 14.08±1.683 | 13.63±1.241 | 13.25±2.478 | 0.5112 |
| Sodium [mmol/l] | 139.5±2.07 | 140.5±2.963 | 142.5±3.214 a | 142.3±1.931 a | 0.0009 |
| Potassium [mmol/l] | 4.014±0.5532 | 4.208±0.5268 | 4.091±0.4724 | 4.333±0.3507 | 0.18 |
| Chloride [mmol/l] | 99.83±2.223 | 99.36±2.423 b | 101.1±2.968 | 101.7±2.786 | 0.0222 |
| Aspartate transaminase [U/l] | 21.04±4.264 | 26.05±9.923 | 25.3±10.99 | 19.4±5.753 | 0.0318 |
| Alanine transaminase [U/l] | 16.21±5.122 | 20.6±8.864 | 17.1±6.569 | 16.15±11.55 | 0.2953 |
| Glucose [mg/dl] | 102.1±9.825 | 111.9±13.95 | 102.6±12.7 | 110.8±14.77 | 0.0264 |
| Creatinine [mg/dl] | 0.7217±0.06877 | 0.793±0.1322 | 0.812±0.1676 | 0.7855±0.1619 | 0.1951 |
| Urea [mg/dl] | 38.02±2.716 | 36.2±12.59 | 43.5±10.73 | 42.15±13.33 | 0.1144 |
| C-reactive protein [mg/l] | 2.31±0.7091 | 3.125±3.135 | 3.365±3.781 | 2.965±3.688 | 0.7478 |
| Total calcium [mEq/l] | 4.37±0.2129 | 4.481±0.6306 | 4.438±0.237 | 4.33±0.2462 | 0.5836 |
| Vitamin B12 [pg/ml] | 390.9±101.1 | 472.6±303.4 | 427.5±416.8 | 487.7±421,5 | 0.7948 |
| Folic acid [ng/ml] | 4.818±2.191 | 5.347±2.548 | 6.021±3.659 | 4,875±1,845 | 0.4632 |
| Thyroid stimulating hormone [uIU/ml] | 1.612±0.5624 | 1.267±0.7511 | 1.869±1,424 | 1.536±1.316 | 0.3761 |

# Supplementary Table 2. Characteristics of groups categorized basing on dementia severity (p<0.005: a – vs C; b – vs MD).

| Parameter | C (N=20) | MD (N=17) | MSD (N=43) | ANOVA p-value |
| --- | --- | --- | --- | --- |
| Age [years] | 74.15±5.706 | 79.35±4.609 a | 77.74±5.819 | 0.0143 |
| Education [years] | 6.894±1.094 | 6.529±2.918 | 6.465±2.947 | 0.8279 |
| MMSE 0 score | 28.1±1.027 | 23.01±2.13 a | 12.45± 5.369 a,b | <0.0001 |
| Leukocytes [10^3^/μl] | 7.513±1.114 | 7.253±1.586 | 7.5±2.312 | 0.8925 |
| Lymphocytes [%] | 25.15±4.899 | 25.42±11.8 | 25.28±8.382 | 0.9952 |
| Monocytes [%] | 6.947±1.47 | 7.906±2.131 | 7.749±3.008 | 0.4244 |
| Neutrophiles [%] | 64.18±5.606 | 56.6±9.926 a | 68.6±7.802 b | <0.0001 |
| Eosynophiles [%] | 1.983±0.08617 | 1.565±1.303 | 1.398±1.021 | 0.0883 |
| Basophiles [%] | 0.4389±0.1111 | 0.4059±0.2609 | 0.3317±0.1942 | 0.1061 |
| Erythrocytes [10^6^/μl] | 4.576±0.5064 | 4.56±0.5505 | 4.557±0.4758 | 0.9908 |
| Hemoglobin [g/dl] | 13.45±0.6836 | 13.81±1.478 | 13.87±1.277 | 0.4313 |
| Hematocrit [%] | 41.95±3.108 | 41.2±5.034 | 41.88±4.161 | 0.8243 |
| Mean corpuscular volume [fl] | 91.82±3.225 | 90.5±4.073 | 91.94±5.618 | 0.5675 |
| Mean cell hemoglobin concentration [g/dl] | 32.06±0.7749 | 33.18±1.257 | 31.94±2.061 b | 0.0338 |
| Red cell distibution width [%] | 14.79±0.9156 | 14.45±1.052 | 15.02±1.55 | 0.3279 |
| Platalets [10^9^/μl] | 248.1±55.48 | 290.2±88.78 | 280.8±108.9 | 0.3311 |
| Mean platelet volume [fl] | 8.089±1.18 | 7.941±1.064 | 8.2±0.8176 | 0.6502 |
| Platelecrit [%] | 0.2625±0.04611 | 0.2248±0.05427 | 0.2273±0.08089 | 0.1324 |
| Platelet distibution width [%] | 13.66±1.099 | 13.75±1.833 | 13.61±1.933 | 0.9582 |
| Sodium [mmol/l] | 139.5±2.07 | 141.8±2.525 | 138.6±21.21 | 0.7685 |
| Potassium [mmol/l] | 4.014±0.5532 | 4.27±0.3951 | 4.187±0.4844 | 0.2506 |
| Chloride [mmol/l] | 99.83±2.223 | 101±2.299 | 100.7±3.067 | 0.4215 |
| Aspartate transaminase [U/l] | 21.04±4.264 | 24.18±10.65 | 23.35±9.131 | 0.4906 |
| Alanine transaminase [U/l] | 16.21±5.122 | 23.41±12.6 a | 17.42±8.169 b | 0.0287 |
| Glucose [mg/dl] | 102.1±9.825 | 124.9±28.09 a | 113.7±21.26 | 0.0055 |
| Creatinine [mg/dl] | 0.7217±0.06877 | 0.7565±0.1271 | 0.8128±0.1599 a | 0.0407 |
| Urea [mg/dl] | 38.02±2.716 | 41.41±12.83 | 40.3±12.47 | 0.6211 |
| C-reactive protein [mg/l] | 2.31±0.7091 | 2.888±4.096 | 4.651±7.277 | 0.2735 |
| Total calcium [mEq/l] | 4.37±0.2129 | 4.421±0.2284 | 4.414±0.4675 | 0.8915 |
| Vitamin B12 [pg/ml] | 390.9±101.1 | 377.5±350.9 | 496.3±387.9 | 0.3282 |
| Folic acid [ng/ml] | 4.818±2.191 | 7.029±3.978 a | 4.732±1.723 b | 0.0056 |
| Thyroid stimulating hormone [uIU/ml] | 1.612±0.5624 | 1.599±0.8853 | 1.54±1.321 | 0.965 |
