# Supplementary material for: Serum Amyloid Biomarkers, Tau Protein and YKL-40 Utility in Detection, Differential Diagnosing, and Monitoring of Dementia
Source: Front Psychiatry. 2021 Sep 13;12:725511. doi: 10.3389/fpsyt.2021.725511 (PMC8473887; doi:10.3389/fpsyt.2021.725511)
Supplement: Supplementary file 2 [file Data_Sheet_2.docx]

Supplementary Material

## Supplementary Table 3. Statistically significant correlations were found between examined parameters. MMSE – Mini Mental State Examination; ALT – alanine aminotransferase, CRP – C-reactive protein; Neu – neutrophiles; Ca – calcium; Na – sodium; Crea – creatinine; Cl – chloride; MCV – mean corpuscular volume; Leu – leucocytes; Lym – lymphocytes; Mono – monocytes; PCT – platalecrit; Ery – erythrocytes; Hgb – hemoglobin; Hct – hematocrit; PLT – platelets; YKL-40 0 – YKL-40 concentration on admission; YKL-40 1 – YKL-40 concentration after 4 weeks of treatment; MMSE 0 – MMSE on admission etc.

| Parameters | R | 95% Cl | p |
| --- | --- | --- | --- |
| YKL-40 0 & YKL-40 1 | 0.461 | 0.2342 to 0.6398 | <0.0001 |
| YKL-40 0 & Aβ1-42/Aβ1-40 0 | 0.288 | 0.03707 to 0.5052 | 0.026 |
| YKL-40 0 & MMSE 0 | -0.614 | -0.7507 to -0.4263 | <0.0001 |
| YKL-40 1 & t-tau 1 | 0.36 | 0.1163 to 0.5622 | 0.005 |
| YKL-40 1 & MMSE 1 | -0.563 | -0.7145 to -0.3601 | <0.0001 |
| YKL-40 1 & ALT | -0.293 | -0.3161 to 0.1896 | 0.023 |
| YKL-40 1 & CRP | 0.29 | -0.2512 to 0.2566 | 0.025 |
| YKL-40 1 & Neu | 0.507 | 0.2861 to 0.6767 | <0.0001 |
| t-tau 0 & MMSE 0 | -0.287 | -0.5041 to -0.03550 | 0.026 |
| t-tau 0 & Ca | -0.317 | -0.5281 to -0.06819 | 0.014 |
| t-tau 0 & Na | -0.46 | -0.6392 to -0.2333 | <0.0001 |
| t-tau 1 & MMSE 1 | -0.287 | -0.5041 to -0.03551 | 0.026 |
| t-tau 1 & Neu | 0.269 | 0.01127 to 0.4929 | 0.041 |
| t-tau 1 & Crea | 0.271 | 0.01818 to 0.4910 | 0.036 |
| Aβ1-40 0 & Aβ1-40 1 | 0.323 | 0.07473 to 0.5328 | 0.012 |
| Aβ1-40 0 & Aβ1-40/Aβ1-42 0 | -0.819 | -0.8881 to -0.7131 | <0.0001 |
| Aβ1-40 0 & Cl | -0.27 | -0.4974 to -0.007351 | 0.044 |
| Aβ1-40/AB1-42 1 & Aβ1-40 1 | -0.778 | -0.2503 to 0.2575 | <0.0001 |
| Aβ1-40 1 & MCV | -0.28 | -0.5021 to -0.02349 | 0.033 |
| Aβ1-40 1 & folic acid | 0.275 | 0.01752 to 0.4976 | 0.037 |
| Aβ1-42 0 & Leu | 0.355 | 0.1069 to 0.5621 | 0.006 |
| Aβ1-42 0 & Lym | -0.307 | -0.5239 to -0.05309 | 0.019 |
| Aβ1-42 0 & Mono | -0.353 | -0.5603 to -0.1044 | 0.007 |
| Aβ1-42 0 & PCT | 0.272 | 0.01517 to 0.4958 | 0.039 |
| Aβ1-42 0 & Cl | -0.295 | -0.5181 to -0.03525 | 0.027 |
| Aβ1-40/Aβ1-42 1 & MCV | -0.083 | 0.009657 to 0.4916 | 0.043 |
| Age & education | -0.339 | -0.5463 to -0.09362 | 0.008 |
| Age & Ery | -0.325 | -0.5385 to -0.07335 | 0.013 |
| Age & Hgb | -0.406 | -0.6013 to -0.1651 | 0.002 |
| Age & Hct | -0.426 | -0.6161 to -0.1879 | 0.001 |
| MMSE 0 & MMSE 1 | 0.747 | 0.6078 to 0.8410 | <0.0001 |
| MMSE 0 & CRP | -0.407 | -0.5992 to -0.1710 | 0.001 |
| MMSE 0 & Neu | -0.674 | -0.7937 to -0.5027 | <0.0001 |
| MMSE 1 & Leu | 0.324 | 0.07165 to 0.5373 | 0.013 |
| MMSE 1 & PLT | 0.318 | 0.06468 to 0.5323 | 0.015 |
| MMSE 1 & MPV | -0.311 | -0.5269 to -0.05720 | 0.018 |

**Supplementary Table 4. ROC analysis of serum amyloid biomarkers compared between mild dementia (MD), moderate to severe dementia (MSD), and control group.**

| Comparison | AUC | 95% Cl | P | Cut-off | Sensitivity% | 95% CI | Specificity% | 95% CI |
| --- | --- | --- | --- | --- | --- | --- | --- | --- |
|  | **Aβ1-40** | | | | | | | |
| C vs MD | 0.7535 | 0.6276 to 0.8794 | 0.0013 | > 176.0 | 67.44 | 52.52% to 79.51% | 70 | 48.10% to 85.45% |
| C vs MSD | 0.8706 | 0.7514 to 0.9897 | 0.0001 | > 188.5 | 82.35 | 58.97% to 93.81% | 85 | 63.96% to 94.76% |
| MD vs MSD | 0.6279 | 0.4790 to 0.7768 | 0.1251 | > 225.1 | 58.82 | 36.01% to 78.39% | 60.47 | 45.58% to 73.63% |
|  | **Aβ1-42** | | | | | | | |
| C vs MD | 0.7709 | 0.6578 to 0.8841 | 0.0006 | > 8.375 | 72.09 | 57.31% to 83.25% | 70 | 48.10% to 85.45% |
| C vs MSD | 0.7265 | 0.5598 to 0.8931 | 0.0189 | > 8.389 | 52.94 | 30.96% to 73.83% | 75 | 53.13% to 88.81% |
| MD vs MSD | 0.5253 | 0.3556 to 0.6951 | 0.7615 | < 8.762 | 52.94 | 30.96% to 73.83% | 53.49 | 38.92% to 67.49% |
|  | **Aβ1-42/Aβ1-40** | | | | | | | |
| C vs MD | 0.6977 | 0.5653 to 0.8300 | 0.0121 | < 0.04810 | 60.47 | 45.58% to 73.63% | 60 | 38.66% to 78.12% |
| C vs MSD | 0.8294 | 0.6917 to 0.9671 | 0.0006 | < 0.04626 | 76.47 | 52.74% to 90.44% | 75 | 53.13% to 88.81% |
| MD vs MSD | 0.6033 | 0.4531 to 0.7535 | 0.2155 | < 0.03832 | 58.82 | 36.01% to 78.39% | 60.47 | 45.58% to 73.63% |

**Supplementary Table 5. Multifactorial regression of the amyloid biomarkers.**

| Dependent variable | Independent variable | | | |
| --- | --- | --- | --- | --- |
|  |  | Age | Sex | MMSE |
| **Aβ1-40** | EE | -1.95 | 0.6206 | 2.101 |
|  | 95%CI | -5.610 to 1.711 | -41.40 to 42.64 | -0.8373 to 5.039 |
|  | P-value | 0.2905 | 0.9765 | 0.1576 |
| **Aβ1-42** | EE | -0.02261 | 0.6098 | 0.009937 |
|  | 95%CI | -0.07917 to 0.03394 | -0.02780 to 1.247 | -0.03762 to 0.05749 |
|  | P-value | 0.4262 | 0.0605 | 0.6768 |
| **Aβ1-42/Aβ1-40** | EE | 0.000954 | 0.004401 | -0.00068 |
|  | 95%CI | -0.0002389 to 0.002148 | -0.009297 to 0.01810 | -0.001633 to 0.000282 |
|  | P-value | 0.1148 | 0.5225 | 0.1632 |
